# Editorial: Digital mental health and wellbeing under crisis

**DOI:** 10.3389/fdgth.2024.1404444

**Published:** 2024-04-29

**Authors:** Koustuv Saha, Kokil Jaidka, Jennifer Kim, Jina Suh

**Affiliations:** ^1^ Department of Computer Science, University of Illinois at Urbana-Champaign, Urbana, IL, United States; ^2^ Department of Communications and New Media, National University of Singapore, Singapore, Singapore; ^3^ School of Interactive Computing, Georgia Institute of Technology, Atlanta, GA, United States; ^4^ Microsoft Research, Redmond, WA, United States

**Keywords:** mental health, wellbeing, digital health, crisis, interventions, marginalized populations, vulnerable populations

**Editorial on the Research Topic** Digital mental health and wellbeing under crisis

Mental health is a growing concern, and external crises, both local and global, can proliferate individual and community-cascading mental health ramifications. It becomes critically important to not only gauge the mental health needs of individuals and communities but also to provide proactive, timely, and tailored support in crisis circumstances. Nevertheless, there exist significant gaps in both contemporary research and practical approaches to effectively address mental health issues, especially during times of crisis. In addition, often, the mental health needs of vulnerable and marginalized populations remain unrealized and unattended. In recent years, the proliferation of digital data footprints across a variety of platforms such as wearables, smartphones, sensors, social media, and the web has presented significant promise in advancing our understanding of mental health and refining intervention strategies (1–5). The ubiquity and extensive adoption of a variety of computing technologies have facilitated the unobtrusive collection of rich and longitudinal data both in real-time and on the scale for deeper insights into the demand for and efficacy of mental health care. Digital platforms have been shown to accelerate the delivery of mental health care and bring affordable and accessible mental health care within reach of the individuals and communities that need it (6). In parallel, researchers have also highlighted the gaps in the literature that pertain to the ethical concerns about gauging mental health from digital traces (7–9).

With the above in mind, this Research Topic aimed to encourage and highlight research on how we can transition into the next phase of digital and technological research and interventions in mental health, with a focus on crises and anomalous circumstances (e.g., natural disasters, pandemics, recession, human-made disasters like gun violence, shooting events, wars, etc.). We welcomed research that studies and promotes the emotional resilience of vulnerable, marginalized, and understudied populations (including, but not limited to, marginalizations based on gender, race, sexual orientation, age, physical attributes, disability, neurodiversity, socio-economic status, etc). Further, we called for research that critiques and discusses ethical practices and provides guidelines for more responsible design and deployment of digital mental health research.

We have six papers published on this research topic, highlighting the challenges faced by crises and anomalous circumstances as well as novel ways to meet those challenges (Table 1).

**Table 1:** Summary of publications in the research topic.

Paper	Type	Problem	Method	Population
Alavi et al.	Original Research	Effectiveness of a mental health intervention (in-person and online CBT)	Large-scale study, non-randomized control trial	Major Depressive Disorder (MDD)
Du	(Opinion) Lit. Review	Strategies employed by social workers during the pandemic	Literature Review	Vulnerable and under-served populations, including but not limited to children and older adults, poor, disabled, and homeless individuals.
Kim et al.	Original Research	Effectiveness of online mindfulness	Quantitative analyses (e.g., regression modeling)	Youth exposed to screen during the pandemic.
Zimmerman et al.	Study protocol	Designing and piloting mental health support	Surveys, semi-structured interviews, co-design, intervention deployment	Young adults in poverty.
Zhu et al.	Protocol	Effectiveness of digital storytelling	Design workshops with multi-stakeholders	Mild Cognitive Impairment (MCI), particularly in low-income countries.
Choi et al.	Original Research	Effectiveness of web application to support mental health	RCT, latent profile analyses	LGBTQ+ youth.

As this research topic was introduced right around the corner of the COVID-19 pandemic, three of the papers focus on mental health challenges induced by the pandemic and faced by both support seekers and support providers.

Alavi et al. focuses on the increased prevalence of mental health disorders during the pandemic that were met with additional challenges of increased inaccessibility to care due to pandemic-related safety measures (Alavi et al.). Such challenges led to the adaptation of in-person therapies into digital format administered through phone, internet or mobile apps. They conducted a large-scale study comparing in-person and online cognitive behavioral therapy (CBT) among patients with major depressive disorders (MDD). The participants (n=108) were given a choice of the kind of CBT, and interestingly, participants who chose in-person CBT had much higher baseline symptomatology scores than the ones who chose e-CBT—revealing that there might be differences in preferences based on different levels of MDD symptoms. Nevertheless, both the groups showed comparable significant improvements—highlighting that e-CBT can be a suitable solution to overcome challenges associated with in-person settings, including accessibility barriers and the time invested in care.

Du, on the other hand, focuses on the challenges faced by the social workers, with the increases risks to the social workers due to the lack of adequate protective gear or contact with infected individuals as well as the inaccessibility to patients due to social distancing measures (Du). Their opinion piece highlights social workers’ ability to combat pandemic’s disruptions to “taken-for-granted” at home visits with creative alternative solutions of facilitating digital intimacy. They conducted a literature review on how the social workers served the vulnerable and under-served populations, including but not limited to children and older adults, poor, disabled, and homeless individuals during the COVID-19 pandemic (Du). The authors note that while the social workers banked on phone and video-call-based solutions to serve in work-from-home settings, the importance of in-person setups was not lost. The social workers planned alternative strategies of in-person meetings with necessary precautions, e.g., meeting older adults on outdoor walks rather than in enclosed home setups (Du).

Kim et al. focused their attention on the youth, whose social-emotional development was disrupted by the pandemic-induced switch to virtual classes and online education and the prolonged screen time. They examined the effectiveness of online mindfulness in building social-emotional competencies, such as resilience, self-esteem, and self-comparison (Kim et al.). The authors focused on a population (n=117) of *youth exposed to screens during the pandemic*. By adopting regression modeling analyses, this paper notes the efficacy of mindfulness and recommends using online mindfulness in building social-emotional competencies.

The COVID-19 pandemic was not the only crisis highlighted in this research topic. Zimmerman et al. direct their attention to mental health concerns, such as post-traumatic stress disorder (PTSD), depression, and anxiety, arising from prolonged exposure to internal armed conflict in Colombia. Similarly to Kim et al., they focus on the youth, but in this case, the youth they target may have experienced loss of family or friends in the armed conflict, witnessed violent events, or be victims of violent themselves. The authors published a study protocol on mental health interventions for young adults in poverty enrolled in post-secondary education in post-conflict regions in Colombia (Zimmerman et al.). The authors propose a study on designing and piloting mental health support interventions embedded within the existing Youth-in-Action program introduced by the Colombian government in 2012 to improve the livelihood of vulnerable young people living in poverty. The study protocol proposes a three-phase approach, including (1) *a pre-pilot survey and semi-structured interviews*, with facilitators and beneficiaries of JeA program, to understand the needs and barriers to mental health interventions, (2) *co-design* sessions with stakeholders to refine and develop the mental health intervention, and (3) *intervention deployment*, when the intervention will be piloted to examine its feasibility, adaptability, efficiency, and usefulness in real settings. Overall, this study will conduct quantitative and qualitative data analyses toward empirically informed strategies to address mental health concerns among socioeconomically vulnerable young populations in low- and middle-income countries.

In addition to addressing mental health challenges faced during crises, this research topic includes two papers that propose human-centered approaches to addressing methodological gaps in supporting vulnerable populations.

Choi et al. challenges the prevailing approach of using individual paradata metrics (i.e., amount, duration, and depth of use) for understanding engagement in digital health interventions (DHIs). Given that it is often hard to assess whether participants received the intended dose of a digital health intervention, Choi et al. conducted a person-centered study to analyze engagement patterns within a randomized control trial (RCT) of *imi*, a web-application to support the mental health of LGBTQ+ youth (Choi et al.). In particular, the authors employed latent profile analysis as a statistical method to classify user engagement and then measure the differences in stress appraisals. For example, a high engagement profile reported significantly higher challenge appraisals than an average engagement profile. The findings of this study support prior findings, provide an approach to mitigate concerns related to multi-collinearity and add nuance and context.

Along similar lines, in another study focusing on mental health interventions in low-income countries, Zhu et al. advocates for placing the experiences of intended users and stakeholders at the center through co-designs. They propose a research protocol on how digital storytelling interventions can enhance social connections and participation for people with mild cognitive impairment (MCI) (Zhu et al.). The authors propose two design workshops with multi-stakeholders of MCI, patients, caregivers, and therapists. This would help build a storytelling application, and then they plan to test its usability and efficacy by conducting a single-blinded field test, evaluating the dimensions of social participation, social connectedness, self-efficacy, happiness, and user experience with the application.

## Potential impacts and future directions

The body of papers on this research topic provides interesting future directions and bears theoretical, practical, and design implications for mental health care in general and for vulnerable populations in particular. The importance of patient-centered approaches to mental health care was noted in multiple studies (Alavi et al., Choi et al.). For instance, with the evolving needs for remote and technology-assisted mediums of mental health interventions, Kim et al. find promising evidence to support the implementation of new forms of mental health interventions that go beyond traditional and in-person settings (Kim et al.). This study also highlights the critical need to cultivate a resilient society, especially amidst times like a global pandemic (Kim et al.). Likewise, Choi et al. emphasize the importance of exploring ways of gaining a comprehensive understanding of user engagement with digital mental health interventions. Alavi et al. noted the need to explore participant perceptions and therapeutic rapport across multiple means of care. Moreover, investigating offline avenues alongside online mental health interventions can offer complementary efficacy (Alavi et al., Zhu et al.).
